# Supplementary material for: Resolving the full spectrum of human genome variation using Linked-Reads
Source: Genome Res. 2019 Apr;29(4):635–45. doi: 10.1101/gr.234443.118 (PMC6442396; doi:10.1101/gr.234443.118)
Supplement: Supplemental Material [file supp_gr.234443.118_Supplemental_longranger-2.2.zip › longranger-2.2.tar.bz2!/lr2.2-public/lib/stan/cmdstan/stan/doc/api/html/check__cholesky__factor__corr_8hpp_source.html]

Stan: src/stan/math/error\_handling/matrix/check\_cholesky\_factor\_corr.hpp Source File


|  |  |
| --- | --- |
|  | Stan  2.5.0  probability, sampling & optimization |


- Main Page
- Related Pages
- Namespaces
- Classes
- Files

- File List
- File Members

All Classes Namespaces Files Functions Variables Typedefs Enumerations Enumerator Friends Macros Pages

- src
- stan
- math
- error\_handling
- matrix

check\_cholesky\_factor\_corr.hpp

Go to the documentation of this file.

1 #ifndef STAN\_\_MATH\_\_ERROR\_HANDLING\_\_MATRIX\_\_CHECK\_CHOLESKY\_FACTOR\_CORR\_HPP

2 #define STAN\_\_MATH\_\_ERROR\_HANDLING\_\_MATRIX\_\_CHECK\_CHOLESKY\_FACTOR\_CORR\_HPP

3

4 #include <stan/math/matrix/Eigen.hpp>

5 #include <stan/math/error\_handling/check\_positive.hpp>

6 #include <stan/math/error\_handling/matrix/check\_lower\_triangular.hpp>

7 #include <stan/math/error\_handling/matrix/check\_square.hpp>

8 #include <stan/math/error\_handling/matrix/constraint\_tolerance.hpp>

9 #include <stan/math/error\_handling/matrix/check\_unit\_vector.hpp>

10

11 namespace stan {

12

13  namespace math {

14

33  template <typename T\_y, typename T\_result>

34  bool check\_cholesky\_factor\_corr(const char\* function,

35  const Eigen::Matrix<T\_y,Eigen::Dynamic,Eigen::Dynamic>& y,

36  const char\* name,

37  T\_result\* result) {

38  if (!check\_square(function,y,name,result))

39  return false;

40  if (!check\_lower\_triangular(function,y,name,result))

41  return false;

42  for (int i = 0; i < y.rows(); ++i)

43  if (!check\_positive(function, y(i,i), name, result))

44  return false;

45  for (int i = 0; i < y.rows(); ++i) {

46  Eigen::Matrix<T\_y,Eigen::Dynamic,1> y\_i = y.row(i).transpose();

47  if (!check\_unit\_vector(function, y\_i, name, result))

48  return false;

49  }

50  return true;

51  }

52

53  template <typename T>

54  inline bool check\_cholesky\_factor\_corr(const char\* function,

55  const Eigen::Matrix<T,Eigen::Dynamic,Eigen::Dynamic>& y,

56  const char\* name,

57  T\* result = 0) {

58  return check\_cholesky\_factor\_corr<T,T>(function,y,name,result);

59  }

60

61

74  template <typename T\_y, typename T\_result>

75  inline bool check\_cholesky\_factor\_corr(const char\* function,

76  const Eigen::Matrix<T\_y,Eigen::Dynamic,Eigen::Dynamic>& y,

77  T\_result\* result) {

78  return check\_cholesky\_factor\_corr(function,y,"(internal variable)",

79  result);

80  }

81

82  template <typename T>

83  inline bool check\_cholesky\_factor\_corr(const char\* function,

84  const Eigen::Matrix<T,Eigen::Dynamic,Eigen::Dynamic>& y,

85  T\* result = 0) {

86  return check\_cholesky\_factor\_corr<T,T>(function,y,result);

87  }

88

89  }

90 }

91 #endif

check\_positive.hpp

constraint\_tolerance.hpp

check\_lower\_triangular.hpp

stan::math::check\_unit\_vector

bool check\_unit\_vector(const char \*function, const Eigen::Matrix< T\_prob, Eigen::Dynamic, 1 > &theta, const char \*name, T\_result \*result)

Return true if the specified vector is unit vector.

**Definition:** check\_unit\_vector.hpp:27

check\_unit\_vector.hpp

stan::math::check\_positive

bool check\_positive(const char \*function, const T\_y &y, const char \*name, T\_result \*result)

**Definition:** check\_positive.hpp:54

Eigen.hpp

stan::math::check\_lower\_triangular

bool check\_lower\_triangular(const char \*function, const Eigen::Matrix< T\_y, Eigen::Dynamic, Eigen::Dynamic > &y, const char \*name, T\_result \*result)

Return true if the specified matrix is lower triangular.

**Definition:** check\_lower\_triangular.hpp:27

stan::math::check\_square

bool check\_square(const char \*function, const Eigen::Matrix< T\_y, Eigen::Dynamic, Eigen::Dynamic > &y, const char \*name, T\_result \*result)

Return true if the specified matrix is square.

**Definition:** check\_square.hpp:24

stan::math::check\_cholesky\_factor\_corr

bool check\_cholesky\_factor\_corr(const char \*function, const Eigen::Matrix< T\_y, Eigen::Dynamic, Eigen::Dynamic > &y, const char \*name, T\_result \*result)

Return true if the specified matrix is a valid Cholesky factor.

**Definition:** check\_cholesky\_factor\_corr.hpp:34

check\_square.hpp


---

[ Stan Home Page ]

*© 2011–2014,
Stan Development Team.*
